# BOD1 Is Required for Cognitive Function in Humans and *Drosophila*

**DOI:** 10.1371/journal.pgen.1006022

**Published:** 2016-05-11

**Authors:** Sahar Esmaeeli-Nieh, Michaela Fenckova, Iain M. Porter, M. Mahdi Motazacker, Bonnie Nijhof, Anna Castells-Nobau, Zoltan Asztalos, Robert Weißmann, Farkhondeh Behjati, Andreas Tzschach, Ute Felbor, Harry Scherthan, Seyed Morteza Sayfati, H. Hilger. Ropers, Kimia Kahrizi, Hossein Najmabadi, Jason R. Swedlow, Annette Schenck, Andreas W. Kuss

**Affiliations:** 1 Department for Human Genetics, Max Planck Institute for Molecular Genetics, Berlin, Germany; 2 Department of Human Genetics, Donders Institute for Brain, Cognition and Behaviour, Radboud university medical center, Nijmegen, Netherlands; 3 Centre for Gene Regulation and Expression, College of Life Sciences, University of Dundee, Dundee, United Kingdom; 4 Department Genetics, Aktogen Limited, University of Cambridge, Cambridge, United Kingdom; 5 Aktogen Hungary Ltd., Bay Zoltán Nonprofit Ltd., Institute for Biotechnology, Szeged, Hungary; 6 Institute of Biochemistry, Biological Research Centre, Hungarian Academy of Sciences, Szeged, Hungary; 7 Department of Human Genetics, University Medicine Greifswald and Interfaculty Institute of Genetics and Functional Genomics, University of Greifswald, Greifswald, Germany; 8 Genetics Research Center, University of Social Welfare and Rehabilitation Sciences, Tehran, Iran; 9 Institut für Radiobiologie der Bundeswehr in Verbindung mit der Universität Ulm, München, Germany; University of Antwerp, BELGIUM

## Abstract

Here we report a stop-mutation in the *BOD1 (Biorientation Defective 1)* gene, which co-segregates with intellectual disability in a large consanguineous family, where individuals that are homozygous for the mutation have no detectable *BOD1* mRNA or protein. The BOD1 protein is required for proper chromosome segregation, regulating phosphorylation of PLK1 substrates by modulating Protein Phosphatase 2A (PP2A) activity during mitosis. We report that fibroblast cell lines derived from homozygous *BOD1* mutation carriers show aberrant localisation of the cell cycle kinase PLK1 and its phosphatase PP2A at mitotic kinetochores. However, in contrast to the mitotic arrest observed in *BOD1*-siRNA treated HeLa cells, patient-derived cells progressed through mitosis with no apparent segregation defects but at an accelerated rate compared to controls. The relatively normal cell cycle progression observed in cultured cells is in line with the absence of gross structural brain abnormalities in the affected individuals. Moreover, we found that in normal adult brain tissues BOD1 expression is maintained at considerable levels, in contrast to PLK1 expression, and provide evidence for synaptic localization of Bod1 in murine neurons. These observations suggest that BOD1 plays a cell cycle-independent role in the nervous system. To address this possibility, we established two *Drosophila* models, where neuron-specific knockdown of BOD1 caused pronounced learning deficits and significant abnormalities in synapse morphology. Together our results reveal novel postmitotic functions of BOD1 as well as pathogenic mechanisms that strongly support a causative role of BOD1 deficiency in the aetiology of intellectual disability. Moreover, by demonstrating its requirement for cognitive function in humans and *Drosophila* we provide evidence for a conserved role of BOD1 in the development and maintenance of cognitive features.

## Author Summary

Intellectual disability (ID) is a form of cognitive impairment characterized by limitations in cognitive functions that manifest as an intelligence quotient (IQ) below 70. ID has a prevalence of 1–3% in the general population and represents a major health-care problem. To understand the functional consequences of causative mutations we study the disease-causing mechanisms of hereditary acquired mutations that result in ID. Here we describe a large family that has a mutation affecting a gene called *BOD1*. Family members who are homozygous for the mutation (i.e. both maternal and paternal copies of the gene carry the mutation) produce no detectable BOD1 protein and suffer from intellectual disability. We have previously shown that BOD1 is a crucial regulator of an important signalling molecule called Protein Phosphatase 2A (PP2A) during cell division. PP2A also has diverse but poorly understood roles in neuronal function. We demonstrate here that Bod1 can regulate PP2A function throughout the cell cycle and also localises to synapses in neurons. To determine if BOD1 deficiency directly affects the structure and function of neurons we targeted the gene in the model fly organism Drosophila Melanogaster. Neuron-specific knockdown caused pronounced learning difficulties and significant abnormalities in synaptic morphology indicating that BOD1 is involved in an evolutionary conserved mechanism crucial for the development of cognitive features.

## Introduction

Intellectual disability (ID) [1] is a form of cognitive impairment, characterized by limitations in mental functioning that manifest as an intelligence quotient (IQ) below 70. ID has an estimated prevalence ranging between 1% and 3% in the general population [2–4]**.** Mutations in more than 750 genes have been identified that cause ID when mutated [5], but particularly autosomal recessive forms of ID (ARID) are poorly understood. Even though the count of genes known to carry ARID causing mutations is now increasing at an accelerated rate because of the recent broad implementation of high throughput sequencing technologies, there are to date still less than 50 genes reported [4]. In order to increase the knowledge about the molecular basis of ARID, we have previously performed autozygosity mapping and mutation screening in a large cohort of Iranian families with a high percentage of consanguinity and identified several loci implicated in non-syndromic ARID [6,7]**.** In addition, we have identified a diversity of ARID genes involved in several physiological pathways, emphasising the genetic heterogeneity of the non-syndromic ARID phenotype [8–15].The diversity of genes involved in the aetiology of ARID also reflects the complexity of the affected organ, i.e. the brain, so that an increase in knowledge about monogenic causes of ID and their functional implications can greatly contribute to a better understanding of the processes involved in the development and maintenance of the brain and its higher cognitive functions.

We report here on a family with 4 female individuals presenting with ID where we found a single homozygous mutation disrupting the *BOD1 (Biorientation Defective 1)* gene. *BOD1* encodes a highly conserved 22 kDa protein required for proper chromosome biorientation [16]. According to the GTEx Portal (http://www.gtexportal.org/home/gene/BOD1; accessed on 10/02/16) *BOD1* mRNA is expressed in the vast majority of investigated tissues. During mitosis BOD1 regulates Protein Phosphatase 2A (PP2A) activity at the kinetochore [17] by specifically binding to and inhibiting PP2A complexes containing the B56 regulatory subunit. PP2A-B56 localises to mitotic kinetochores during mitosis and controls both kinetochore microtubule attachment and checkpoint signalling [18–22]. Depletion of BOD1 from HeLa cells results in a loss of inhibition of PP2A-B56 and subsequent increase of phosphatase activity at the kinetochore. In particular, BOD1 depletion leads to reduced phosphorylation of PBIP/CENP-U, which results in a failure to recruit the mitotic Polo-Like Kinase 1 (PLK1) [MIM 602098] to kinetochores [8].

Additionally, BOD1 may have other functions in cell and organism physiology. For example, somatic deletions in *BOD1* were previously found in non-pyramidal neurons and cells in white matter from patients with Schizophrenia [23]. Moreover, it has recently been described to interact with the SET1/MLL (SET Domain Containing 1A/Mixed-Lineage Leukemia) complex, a member of the COMPASS-like H3K3 histone methyltransferase multi-subunit complexes. To date, no defects in histone methylation have been linked to BOD1. However, SET1/MLL also contains HCFC1 (Host Cell Factor C1) [MIM 309541] [24], a protein previously implicated in X-linked ID [25,26]**.**

In this report, we describe the consequences of BOD1 deficiency using cell lines derived from fibroblasts of affected individuals. We found that these show changes in PLK1 protein levels, function and mislocalization of PLK1 and PP2A but, unexpectedly, with no associated mitotic impairments. This observation, which is in agreement with an absence of microcephaly in individuals with BOD1 mutations, raised the possibility of a so far unidentified, cell cycle-independent role for BOD1. In support of this hypothesis we provide evidence for a presynaptic localization of BOD1 in mammalian neurons and show that neuron-specific knockdown of the *Drosophila* ortholog of *BOD1* leads to abnormal learning and affects synaptic morphology. Taken together, our findings strongly support the causative role of the *BOD1* mutation in the individuals affected by ID, uncover novel aspects of BOD1 function and pathogenic mechanisms and highlight an evolutionarily conserved role of BOD1 in cognition.

## Results

### A nonsense mutation in *BOD1* co-segregates with ID in a consanguineous Iranian family with four affected individuals

**Fig 1:**
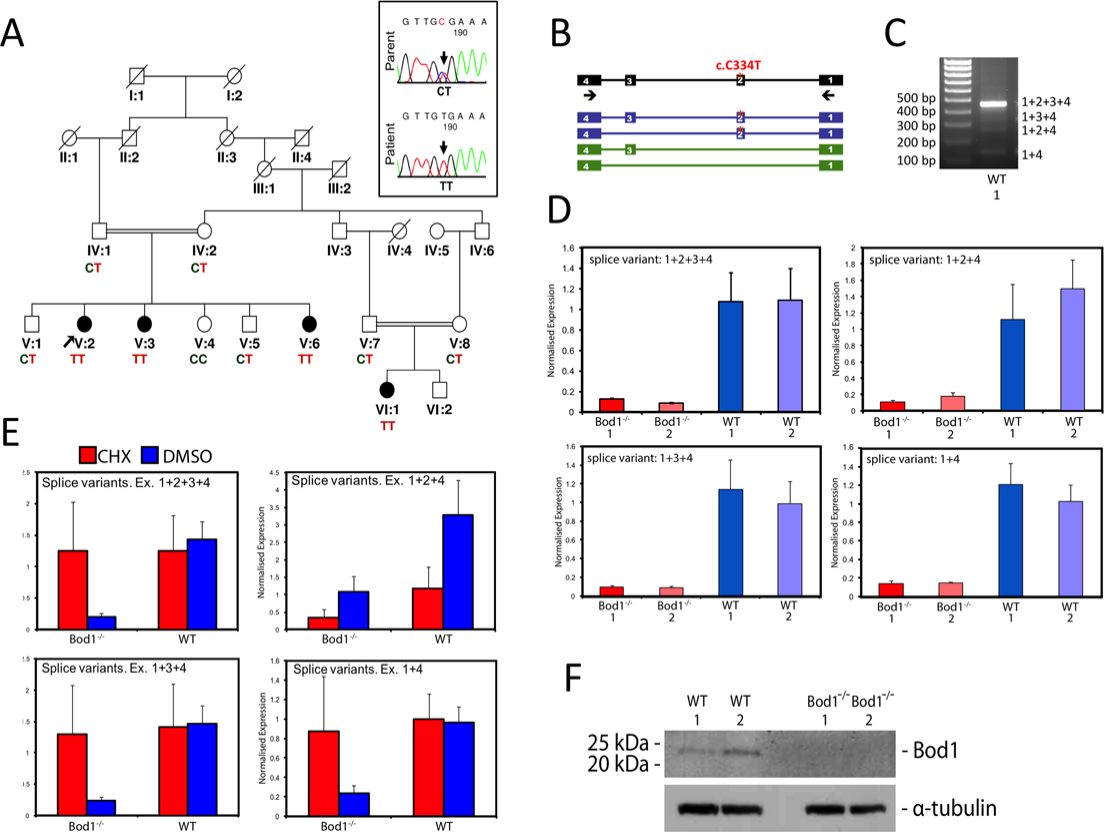
Nonsense Mutation in *BOD1* co-segregates with Intellectual Disability and leads to loss of BOD1 in patient tissues. (A) Family pedigree and co-segregation of the mutation within the family. Filled symbols represent affected individuals. Sequence chromatograms from one patient (V:2) and one parent (IV:1) are shown on the upper right. (B) Schematic representation of BOD1 (black) and the exon composition in alternative transcripts. Previously unknown transcripts are shown in green. Arrows indicate the location of primers used for RT-PCR experiments (C) Agarose Gel electrophoresis results of RT-PCR experiment. (D) qRT-PCR was performed on patient and control Fibroblasts. The experiments were performed twice with independent cells, each time in triplicate (Error bars represent the SEM). One representative result is shown. (E) NMD analyses of patient fibroblasts were performed twice with independent cell samples, each time in triplicate. The results are from pooled patient (*BOD1*^-/-^) and control (WT) samples. CHX: cycloheximide, DMSO:Dimethyl sulfoxide. Error bars represent the SEM. (F) Western blot of protein extracts from fibroblast cells using a Bod1 polyclonal antibody. The Bod1 antibody recognizes a 22KDa protein, matching the full-length Bod1 protein. Alpha tubulin was used as a loading control.

In a family with 4 female individuals with ID (**Fig 1A**) we performed multipoint linkage analysis based on the assumption of an autosomal recessive pattern of inheritance and a disease allele frequency of 0.001. We identified a single 4.3 Mbp interval on chromosome 5q (5q35.1–35.2) with a LOD score of 4.4 (**S1 Fig**) and sequenced the coding regions of all protein coding genes within the interval. This revealed a homozygous point mutation (NM_138369.2:c.334C>T; p.R112X) in the second exon of the *BOD1* gene, which co-segregated with the disease (**Fig 1A**). The mutation was not found in 380 Iranian and 340 German control chromosomes and was absent in 200 Danish exomes [27]. In addition, the NM_138369.2:c.334C>T mutation was not found in the current data release (ESP6500SI-V2) of the Exome Variant Server (http://evs.gs.washington.edu/EVS/), NHLBI GO Exome Sequencing Project (ESP), Seattle, WA (accessed June 2015), containing exome sequencing results from 6503 individuals, nor in data from the 1000 Genomes Project [28], nor in the Exome sequencing Results from 60,706 unrelated individuals compiled by the Exome Aggregation Consortium (ExAC), Cambridge, MA (http://exac.broadinstitute.org, accessed February 2016). Moreover, our sequencing of controls and database search also revealed no other homozygous deleterious mutations in other parts of the *BOD1* coding region.

The three affected females of the left branch of the family pedigree (V:2; V:3; V:6) suffered from moderate ID with an IQ of 50–55 (determined by Wechsler’s scale) in all three cases. In addition, these individuals presented with either primary (V:3) or secondary (V:2; V:6) amenorrhoea of unknown cause. Endocrinological tests and ultrasound investigations of the ovaries revealed no abnormalities. The affected individual in the right branch of the family pedigree (VI:1) presented with mild ID (IQ: 70–75). Brain MRI scans were performed on all four affected individuals, but revealed no consistent morphological abnormalities. All four individuals were obese or overweight but this phenotype did not co-segregate with the BOD1 mutation. From patient V:2 we obtained a lymphoblast cell line (LCL). In addition we were able to establish fibroblast cell lines from affected individuals V:2 and V:3. Cells derived from homozygous mutation carriers will be referred to as *BOD1*^*-/-*^ cells throughout the manuscript.

RT-PCR and sequencing analyses of control fibroblasts showed the presence of the full length *BOD1* transcript (NM_138369.2), comprising all exons (1–4), represents the main isoform of the protein, which is 185 amino acids long and most likely its dominant functional form. In addition we detected three comparatively weakly expressed additional transcripts (**Fig 1B and 1C**), composed of the exons 1+3+4 (isoform b, NM_001159651.1, comprising 129 amino acids), 1+2+4 (ENST00000285908.5, encoding 129 amino acids) and 1+4 (ENST00000480951, encoding 85 amino acids). Quantitative PCR analyses of RNA preparations from control and *BOD1*^*-/-*^ cell lines further revealed that *BOD1* mRNA was absent in cell lines from both affected individuals (**Fig 1D**). This loss of BOD1 mRNA is likely caused by nonsense mediated decay (NMD) as mRNA levels of 3 of the 4 detected splice variants were increased to near-control levels following treatment with cycloheximide (**Fig 1E**). To confirm the loss of the main BOD1 isoform at the protein level, we investigated the patient cell lines by western blot, using a rabbit polyclonal antibody raised against recombinant full length GST-BOD1 [16]. In keeping with our quantitative PCR-results, this isoform was not detected in either *BOD1*^*-/-*^ cell line (**Fig 1F**).

### *BOD1*^-/-^ fibroblasts progress through mitosis at an accelerated rate

**Fig 2:**
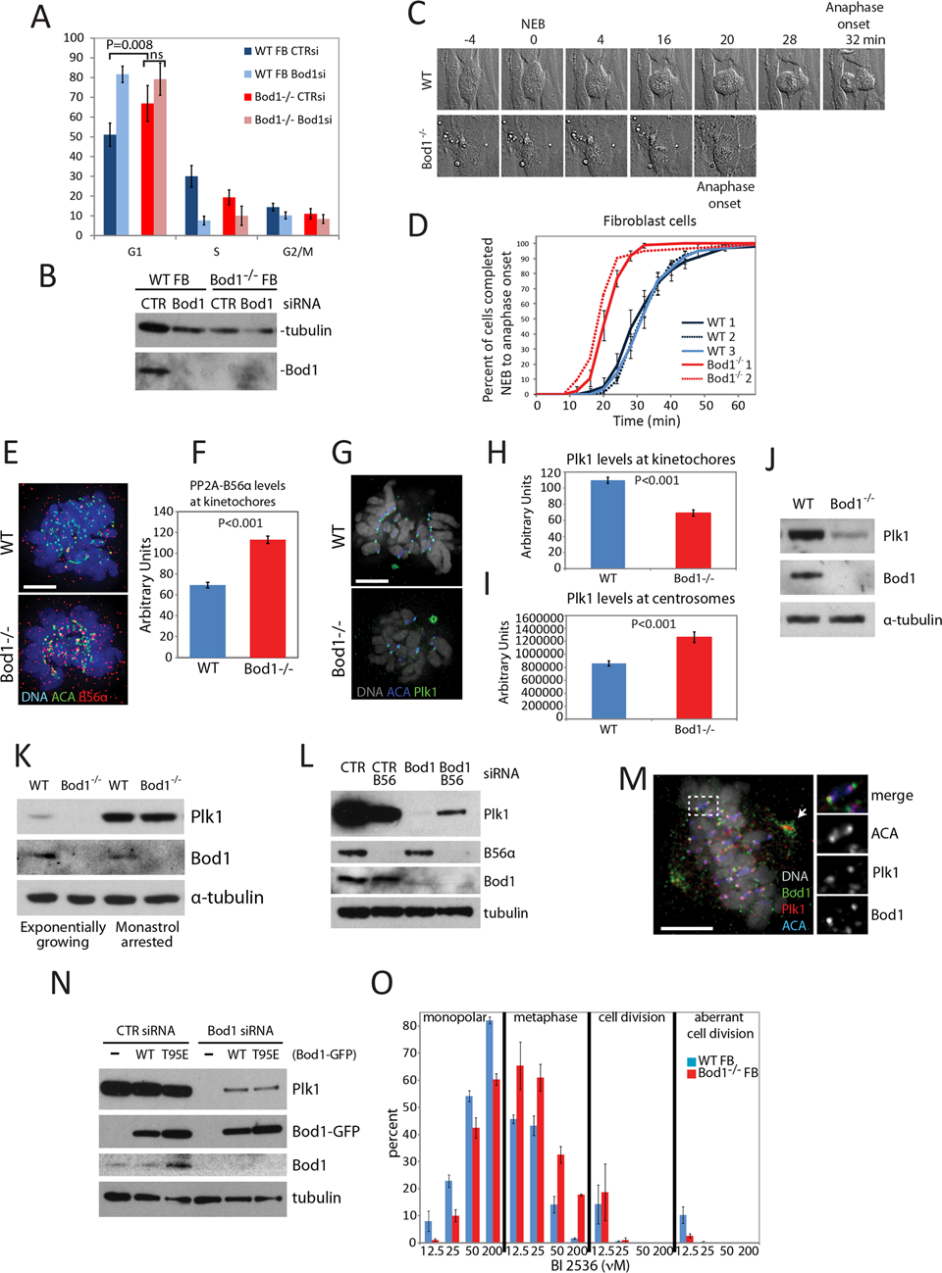
Functional consequences of the absence of BOD1 in patient-derived fibroblasts. (A) Flow cytometric analysis of cell cycle profile in WT and *BOD1*^-/-^ primary fibroblasts electroporated with control or *BOD1* siRNA. Error bars represent standard deviation. (B) Immunoblotting of BOD1 and tubulin from cell lysates simultaneously electroporated with samples analysed in (A). (C) Representative DIC timelapse imaging of primary fibroblast cells undergoing mitosis. Nuclear Envelope Breakdown (NEB) and Anaphase Onset (AO) are indicated. (D) Cumulative timing of NEB to AO timing in Primary Fibroblast cell lines. Error bars represent standard deviation. P<0.001 for *BOD1*^-/-^ cells to each WT sample. Insufficient data collected for *BOD1*^-/-^ cells to determine statistical significance. (E) Immunofluorescence localization of PP2A-B56 in WT and *BOD1*^-/-^ Primary fibroblasts. DAPI (blue), centromeres (detected with ACA) (green), anti-PP2A-B56α (red). (F) Mean B56α levels at kinetochores of WT and *BOD1*^-/-^ Primary fibroblasts (P<0.001). Error bars represent SEM. (G) Immunofluorescence localization of PLK1 in WT and *BOD1*^-/-^ Primary fibroblasts. DAPI (white), anti-PLK1 (green), ACA (blue). (H-J) Mean PLK1 levels at kinetochores and centrosomes of WT and *BOD1*^-/-^ Primary fibroblasts, respectively (P<0.001 in each instance). Error bars represent SEM. (K) Immunoblotting of PLK1, BOD1 and tubulin in asynchronous WT and *BOD1*^-/-^ primary fibroblasts. (L) Immunoblotting of PLK1, BOD1 and tubulin in asynchronous and Monastrol arrested WT and *BOD1*^-/-^ Primary fibroblasts. (M) Immunofluorescence localization of Bod1 in WT Primary Fibroblasts. DAPI (white), ACA (blue), anti-Plk1 (red), anti-Bod1 (green). Scale = 5 μm. Inset shows a single bioriented kinetochore pair. (N) Immunoblotting of PP2A-B56δ, PLK1 and tubulin in WT primary fibroblast electroporated with indicated combinations of CTR, B56-pool or *BOD1* siRNA. Rescue of WT primary fibroblasts after siRNA depletion of Bod1 with plasmids expressing GFP fused to either siRNA resistant WT Bod1 or Bod1^T95E^. (O) Mitotic profile of WT primary fibroblasts and *BOD1*^-/-^ fibroblasts 1 hr after release from RO 3306 into the indicated concentrations of BI 2536. Results show average of three independent experiments. A minimum of 100 mitotic cells counted per condition per experiment. Error bars represent SEM.

We have previously reported that siRNA-mediated knockdown of BOD1 in HeLa cells produces profound chromosome biorientation defects and a block in mitotic segregation [16]. We therefore set out to determine whether abnormalities in cell cycle progression occur in cell lines derived from *BOD1*^-/-^ individuals. We first examined cell cycle progression in WT and *BOD1*^-/-^ fibroblast cells (**Fig 2A**). This revealed a significant increase in the G1 population in *BOD1*^-/-^ cells compared to WT. Treating WT fibroblasts with *BOD1* siRNA resulted in a similar accumulation of cells in G1 (**Fig 1A and 1B**) suggesting this change in cell cycle distribution is directly due to loss of BOD1. We observed no mitotic figures in WT fibroblast cells depleted of BOD1 by siRNA, suggesting that any cells with fully replicated DNA in Fig 2A were in G2. In contrast, mitotic cells could be observed in *BOD1*^-/-^ cells suggesting these cells are capable of progressing through the cell cycle, but with a delay in progressing out of G1.

To detect any gross defects in mitotic chromosome segregation, we next examined the mitotic timing of *BOD1*^-/-^and control WT fibroblasts using DIC time-lapse microscopy, measuring the time from nuclear envelope breakdown (NEB) to anaphase onset (**Fig 2C; see Methods**). *BOD1*^-/-^ fibroblasts progressed through mitosis rapidly, with 50% of cells completing mitosis in 20 min compared to 30 min for the control cells (**Fig 2D**). Similar results were obtained for *BOD1*^-/-^ LCL cells (**S2A Fig**).

Examination of fixed *BOD1*^-/-^ fibroblasts by immunofluorescence revealed no significant increase in cells with unaligned or malformed spindles (**S2B Fig**), suggesting only a subtle disturbance of mitotic regulation in these cells. This is surprising given the profound biorientation defects observed in BOD1-depleted HeLa cells. We therefore explored the properties of mitotic *BOD1*^*-/-*^ cells in more detail.

### *BOD1*^-/-^ fibroblasts show mislocalisation of PLK1 and PP2A

Bod1 is required for proper chromosome alignment during mitosis and the proper phosphorylation of several substrates of the PLK1 and Aurora B (AURKB [MIM 604970]) protein kinases. This effect occurs through the modulation of PP2A-B56 activity [16]. To determine if these pathways were affected in *BOD1*^*-/-*^ cell lines, we investigated the localisation of PP2A-B56 and PLK1. In agreement with previously published observations in BOD1-depleted HeLa cells, *BOD1*^*-/-*^ fibroblasts had increased levels of PP2A-B56 at kinetochores (**Fig 2E and 2F**). Furthermore, PLK1 levels were reduced at the kinetochores of *BOD1*^-/-^ cells compared to control fibroblasts (**Fig 2G–2I**), just as in Bod1-depleted HeLa cells. However, unlike BOD1 depleted HeLa cells, *BOD1*^*-/-*^ fibroblasts had increased PLK1 concentrations at centrosomes.

We also observed that total PLK1 protein levels were reduced in asynchronous *BOD1*^*-/-*^ fibroblasts (**Fig 2J).** However, when these cells were synchronised in mitosis using the Eg5 inhibitor Monastrol they exhibited PLK1 levels that were comparable to control fibroblasts (**Fig 2K)**.

To determine if the changes in protein stability of PLK1 were limited to *BOD1*^*-/-*^ cell lines, we performed siRNA-mediated knock down of BOD1 in wild-type primary fibroblasts. The reduction in PLK1 levels was even more pronounced than that observed in *BOD1*^*-/-*^ cells with little or no detectable PLK1 protein (**Fig 2L**). PLK1 protein levels were partially recovered by co-depletion of BOD1 and PP2A-B56 (**Fig 2L**) suggesting that Bod1 inhibits PP2A mediated PLK1 destabilisation and that PP2A regulates PLK1 function throughout the cell cycle.

Since WT fibroblasts depleted of BOD1 do not enter mitosis, we next tested whether BOD1 has any role in mitosis in WT primary fibroblasts. We used immunofluorescence to localise endogenous Bod1 and observed prominent localisation to mitotic centrosomes and kinetochores (**Fig 2M**), suggesting that a mitotic function for Bod1 is present in these cells, but is masked in siRNA studies by an additional pathway in primary fibroblasts that results in G1 arrest. This G1 pathway is likely lost in highly transformed HeLa cells. To ensure the arrest was not limited to fibroblast cells, we depleted Bod1 from immortalised, non-transformed RPE1 cells and also observed a G1 arrest (**S2C Fig**). Once more, localisation of BOD1 to kinetochores and centrosomes was observed in untransfected RPE1 cells (**S2D Fig**), suggesting multiple roles for BOD1 throughout the cell cycle.

To check the specificity of the Bod1 siRNA, we rescued Bod1 depletion with plasmids expressing WT or constitutively active Bod1 [17] and observed rescue of PLK1 protein levels (**Fig 2N**) confirming the specificity of the effect on PLK1 by Bod1 siRNA. We conclude that BOD1 depletion in primary cells causes loss of PLK1 and that BOD1^-/-^ fibroblasts stabilise sufficient Plk1 to successfully progress through the cell cycle.

### Changes in PLK1 function in *BOD1*^*-/-*^ cells

Since *BOD1*^*-/-*^ cells are able to propagate and progress through the cell cycle, we hypothesised that they must have modulated the function of PLK1 to adapt to the presence of reduced PLK1 levels during interphase (**Fig 2J**). To test this hypothesis, we arrested cells at the G2/M boundary, where PLK1 is required for progression into M phase using RO3306, a CDK1 inhibitor. We then released cells into fresh medium containing increasing amounts of the specific PLK1 inhibitor BI2536. This assay uses the formation of bipolar mitotic spindles as a reporter of PLK1 function [17]. Control WT fibroblasts exposed to BI2536 showed a reduced number of bipolar metaphase spindles and an increased number of monopolar spindles as the concentration of BI2536 was increased (**Fig 2O**). By contrast, *BOD1*^*-/-*^ cells showed fewer monopolar spindles and only a small reduction in the frequency of bipolar spindles even at the highest concentrations of BI2536. This result suggests that *BOD1*^*-/-*^ cells are hyposensitive to PLK1 inhibition relative to WT controls and that they have adapted to loss of BOD1 by either increasing or bypassing PLK1 function. Consistent with the former hypothesis, *BOD1*^*-/*-^ cells have increased levels of PLK1 at centrosomes (**Fig 2G and 2I**), a critical location for PLK1 function in centrosome and spindle maturation.

These results suggest that despite an overall reduction in PLK1 levels in *BOD1*^*-/-*^ cells, they have nonetheless adapted and progress through mitosis in a relatively normal manner. This conclusion is in line with our observation that homozygous mutation carriers do not show any gross developmental or structural brain abnormalities, as are often found in individuals affected by ID with mutations in proteins required for centrosome positioning and localisation (see e.g. Chavali et al. [29] or Kuijpers & Hoogenraad [30]). We therefore hypothesized that cognitive impairment caused by BOD1 deficiency might result from the disturbance of a different, possibly brain-specific functional aspect of BOD1.

### BOD1 expression is maintained in postmitotic brain tissues

**Fig 3:**
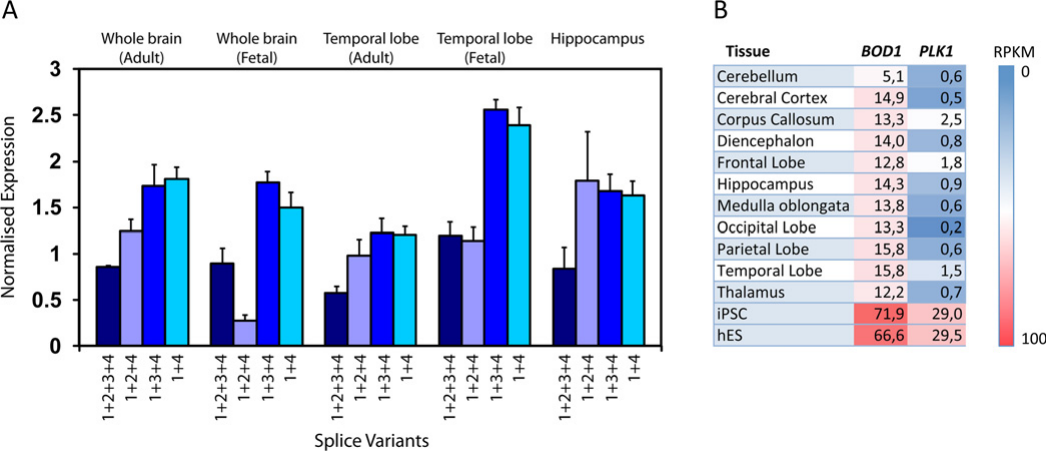
Expression of BOD1 and PLK1 in human tissues. (A) BOD1-specific quantitative RT-PCR experiments were carried out in triplicates, using RNA from the indicated tissues. All splice variants (indicated by the respective exon combinations) were investigated. Error bars represent the SEM. (B) Expression levels i.e. reads per kilobase of transcript per million reads mapped (RPKM), corresponding to BOD1 (NM_138369.2) and PLK1 (NM_005030.5) obtained by RNA-Sequencing of commercially available RNA-samples from different brain tissues, induced pluripotent stem cells (IPSC) and human embryonic stem cells (hES).

Quantitative PCR of RNA from different regions of normal human fetal and adult brain showed that all four BOD1 splice variants detected in lymphocytes and fibroblasts are present throughout the brain, at both investigated developmental stages (**Fig 3A**).

To address whether BOD1 might exert functions independent from cell-cycle regulation of PLK1, we compared BOD1 and PLK1 expression in different areas of the human brain by RNA-sequencing. In agreement with the low rates of dividing cells in these terminally differentiated tissues our results show that PLK1 expression drops to very low levels (**Fig 3B**) as compared to the expression rates observed in pluripotent cells. BOD1 expression, however, is maintained in most investigated brain tissues at about 20% of the expression levels observed in pluripotent cells, strongly suggesting that BOD1 plays a PLK1- and cell cycle-independent role in postmitotic brain cells.

### BOD1 shows synaptic localization in mammalian neurons

**Fig 4:**
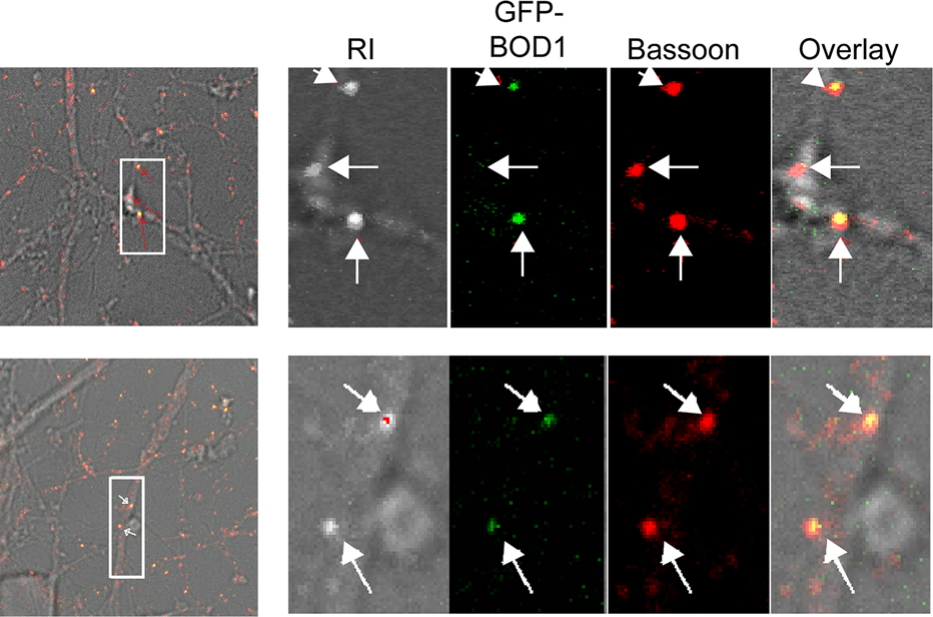
Presynaptic localisation of BOD1 in murine corticoneuronal cells. Representative indirect immunofluorescence confocal image (LSM510) of mouse cortical neurons transfected at day 7 after preparation with 0.1μg BOD1-GFP for 7hrs. Arrows indicate co-localization of BOD1-GFP (in green) with the (pre)synaptic marker anti-Bassoon (red). Insets are magnifications of the boxed area. The range indicator (RI) shows that the images are not overexposed.

Continuing on from the gene expression analysis we were interested in determining BOD1 localisation in mammalian neurons. We analysed murine corticoneuronal cells that were transfected with BOD1-GFP,at short incubation time (7h, to avoid overexpression artefacts). We observed a striking punctuate pattern with BOD1-GFP that co-localised with the synaptic marker Bassoon (**Fig 4**), which suggests a potential role for Bod1 in synaptic signalling.

### BOD1 is required in postmitotic neurons for learning

**Fig 5:**
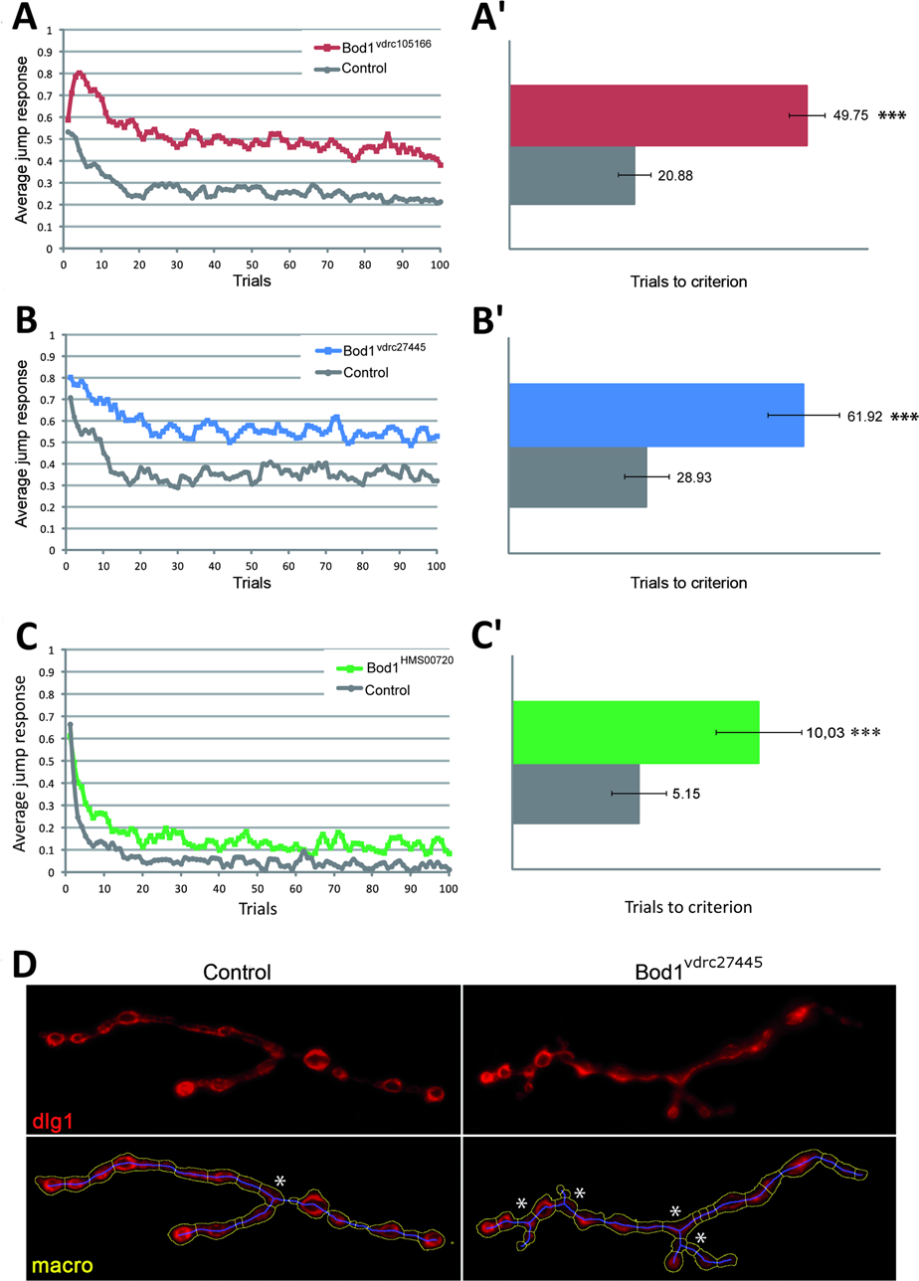
Neuronal knockdown of Drosophila Bod1 affects learning and synapse development. (A-B') Knockdown of *Drosophila* Bod1 using the postmitotic, pan-neuronal promoter elav-Gal4 and three inducible RNAi lines affects non-associative learning in the light-off jump habituation paradigm. Jump responses were induced by repeated light-off pulses for 100 trials with a 1s inter-trial interval. Bod1 knockdown flies of genotypes (A) UAS-Bod1^vdrc105166^/2xGMR-wIR; elav-Gal4, UAS-Dicer2/+, plotted as red squares, (B) UAS-Bod1^vdrc27445^/2xGMR-wIR; elav-Gal4, UAS-Dicer2/+, plotted as blue squares, and (C) 2xGMR-wIR/+; UAS-Bod1^HMS00720^/elav-Gal4, UAS-Dicer2, plotted as green squares, failed to habituate, i.e. to efficiently reduce their jump response upon repeated stimulation. The genetic background controls, generated by crossing the driver line to the respective genetic background of the RNAi line, are shown as grey circles (2xGMR-wIR/+; elav-Gal4, UAS-Dicer2/+). (A', B', C’) Quantification of average jump responses revealed that all three mutant genotypes habituated significantly slower (*** p<0,001). Red bar in (A') Bod1^vdrc105166^, TTC = 49.75, n = 143 versus controls: TTC = 20.88, n = 134. Blue bar in (B'): Bod1^vdrc27445^, TTC = 61.92, n = 93 versus controls TTC = 28.93, n = 87. Green bar in (C)’ Bod1^HMS00720^, TTC = 10.03, n = 70 versus controls TTC = 5.51, n = 68. (D) Knockdown of *Drosophila* Bod1 using the elav-Gal4 promoter and RNAi lines Bod1^vdrc27445^ and Bod1^vdrc105166^ consistently affects synaptic branching at the *Drosophila* Neuromuscular Junction (see text). L3 muscle 4 synapses were labelled with anti-dlg1 and quantified using an in house-developed macro. A Bod1^vdrc27445^ (UAS-Bod1^vdrc27445^/2xGMR-wIR; elav-Gal4, UAS-Dicer2/+) and control (2xGMR-wIR/+; elav-Gal4, UAS-Dicer2/+) synapse is shown. Top panel in red: dlg1 labelling; bottom panels show the macro-annotated, quantified synapse). Asterisks highlight the increased number of synaptic branching points at the mutant synaptic terminal.

To experimentally address whether BOD1 is required directly in neurons for cognitive processes, we took advantage of established animal model for ID disorders, the fruit fly *Drosophila melanogaster*. The *Drosophila* genome encodes a single gene representing the human BOD1 protein family (BOD1, BOD1L1 and BOD1L2), termed CG5514. A multiple sequence alignment is shown as supplemental S3 Fig. From now on we refer to this so far uncharacterized *Drosophila* gene as Bod1. We used the UAS-Gal4 system [31], the panneuronal promoter elav-Gal4 and three inducible RNAi lines carrying different constructs (Bod1^vdrc101981^ Bod1^vdrc4542^, and Bod1^HMS00720^) to knockdown Bod1 specifically in postmitotic neurons. We subjected Bod1 panneuronal knockdown flies to a simple non-associative learning assay: the light-off jump reflex habituation paradigm. In this paradigm, which has previously uncovered learning defects in several *Drosophila* models of Intellectual Disability [32–34] and in classic learning and memory mutants [35], flies are exposed to a repeated light off stimulus at 1 second intervals. Wildtype (wt) flies quickly adapt to the repeated stimulus and gradually suppress their initial jump response as a result of non-associative learning. In our experiments, flies of all tested genetic conditions showed high jump response towards the initial light-off stimulus, demonstrating that they properly perceived the stimulus and that their startle response was not compromised. We found that Bod1 knockdown flies failed to habituate to the presented light-off stimuli compared to their genetic background controls with normal Bod1 levels, and kept on jumping at high levels throughout the entire course of the experiments (**Fig 5**). This learning defect was consistent for two Vienna Drosophila Resource Center (VDRC) RNAi constructs, as well as for a non-overlapping independent short siRNA TriP construct (**Fig 5A, 5B and 5C**). To assess the significance of the habituation defects in both Bod1 knockdown models, flies were considered to have habituated once they failed to jump in five consecutive trials (no-jump criterion). Habituation was quantified as the number of trials required to reach the no-jump criterion (TTC, Trials to criterion). TTC of the Bod1 knockdown flies was 2.38–fold (Bod1^vdrc1105166^, n = 143, p<0.001), 2.14-fold (Bod1^vdrc27445^, n = 93, p<0.001) and 1.95-fold (Bod1^HMS00720^, n = 70, p<0,001) increased over their respective controls (**Fig 5A’, 5B’ and 5C**), revealing significant habituation defects in all three Bod1 *Drosophila* models.

The efficacy of the utilised RNAi constructs was confirmed by qPCR on RNA isolated from *Drosophila* 3^rd^ instar larvae upon ubiquitously-induced knockdown. As Bod1^vdrc27445^ and Bod1^vdrc105166^ contain almost identical siRNA hairpins, only Bod1^vdrc105166^-mediated knockdown was assessed and revealed 23% remaining gene expression; p = 0.0012, student’s t-test; supplemental S1 Table. *Bod1*^*HMS00720*^-mediated RNAi reduced levels of *Bod1* to a lesser but still very significant extend, resulting in 37% remaining gene expression (p = 0.0086, student’s t-test; S1 Table).

We conclude that conditional knockdown of Bod1, induced specifically in neurons, does not affect the overall startle response/acute fitness of the flies but leads to specific and highly consistent learning defects.

Synapse biology is crucial for learning and has been proposed to play a central role in ID [36–38]. Therefore, and because of the observed Bod1 localisation to synapses in cultured neurons, we investigated the consequences of Bod1 knockdown on synapse development. The *Drosophila* larval Neuromuscular junction (NMJ) was investigated in elav-Gal4 induced panneuronal Bod1 knockdown larvae using an antibody against dlg1 that visualizes the overall morphology of synaptic terminals. Knockdown of Bod1 with both Bod1^vdrc27445^ and Bod1^vdrc105166^ lines induces modest but highly significant abnormalities in synapse branching (Fig 5D)The average number of branching points was 2.7 versus 1.6 in the appropriate genetic background control (Bod1^vdrc105166^ versus control); p<0.00001) and 2.5 versus 1.3 (Bod1^vdrc27445^ versus control; p<0.005). Other synaptic parameters such as length or perimeter of the synaptic terminal were not consistently changed. There was no significant NMJ defect in the weaker Bod1^HMS00720^ knockdown condition (1.5 in Bod1^HMS00720^ versus 1.4 in control). We conclude that Bod1 is required for habituation and may control synaptic branching.

## Discussion

We report that BOD1, a protein that has previously been linked to mitotic cell division, is required for cognitive functions in humans and *Drosophila*. This is in line with a previous report that described somatic deletions in BOD1 within non-pyramidal neurons and cells in white matter from patients with Schizophrenia, implicating BOD1 as a player in brain development and, more importantly, in a neuropsychiatric context [23].

In agreement with the absence of gross morphological brain abnormalities in homozygous mutation carriers, our experiments, using neuronal cultures and *Drosophila* as a model organism, support a novel postmitotic function for Bod1 and raise the possibility that abnormal synaptic development or function contributes to cognitive impairment in individuals with *BOD1* mutations.

In the consanguineous family we describe here, a nonsense mutation in exon 2 of *BOD1* co-segregates with moderate-to-severe intellectual disability. This mutation is absent in ethnically matched controls and in all presently accessible genome or exome sequencing data repositories. Furthermore, it is of note that in the ExAC database, which contains exome sequencing data from 60,706 unrelated individuals, only 6 coding positions within BOD1 showed deleterious alterations, of which none was observed in a homozygous state and four were found only once. This shows that homozygous deleterious mutations affecting *BOD1* are not tolerated in intellectually healthy individuals and strongly supports our conclusion that loss of BOD1 function is detrimental to molecular pathways involving this protein and is thus causative for ID.

All four homozygous mutation carriers lack detectable levels of BOD1, presumably because the mutation leads to NMD of the corresponding mRNA. Originally BOD1 was identified as a centrosomal and kinetochore protein that is required for proper chromosome biorientation in HeLa cells [16]. It has previously been shown that BOD1 is involved in the regulation of AuroraB kinase dependent phosphorylation of Mitotic Centromere Associated Kinesin alias Kinesin Family Member 2C (MCAK alias KIF2C [MIM 604538]) [16]. Recently BOD1 has also been shown to act as an inhibitor of PP2A-B56 function at the kinetochore and to regulate the recruitment of a number of proteins to the kinetochore and centrosome, including PLK1 and PP2A [17].

A considerable number of other centrosomal proteins or proteins required for centrosome positioning and localisation have already been associated with ID, including ASPM [39], the human ortholog of the abnormal spindle gene (asp) in *Drosophila* [40], which is essential for the normal functioning of mitotic spindles [41]. Also defects in *MCPH1* [MIM 607117], *CENPJ* [MIM 609279] and *CDK5RAP2* [MIM 608201] have been shown to cause ID (for review see Chavali et al. or Bond et al. [29,42]). However, mutations in these genes usually induce not only cognitive defects but also entail severe brain abnormalities such as primary microcephaly. It is therefore remarkable that homozygous mutation carriers (despite the apparent absence of the main BOD1 transcript) do not show microcephaly or any other gross structural brain abnormalities. Therefore, as *BOD1*^*-/-*^ fibroblasts still proliferate and pass through mitosis, they must have in some way adapted to the loss of BOD1 and to BOD1-mediated PLK1 function, demonstrating a surprising adaptability in the cell cycle control machinery and thus providing an explanation for the absence of structural defects in the patient’s brains. In this context it is interesting that *BOD1*^*-/-*^ *fibroblasts* show an increased tolerance to the PLK1 inhibitor BI2536, which might be explained by the increased localisation of PLK1 to the centrosomes that we observed. Increased PLK1 at centrosomes may stabilize spindles allowing mitosis to proceed and preventing structural deficits in the affected tissues.

BOD1 is still expressed throughout adult human brain tissues, in contrast to the cell cycle kinase PLK1 (Fig 3). This suggests that BOD1 exerts additional functions in mature neurons, and our observation that Bod1 localises to synapses further supports this notion.

In order to further investigate whether and how loss of BOD1 may affect cognitive function independent from its mitotic role in cell cycle progression we established *Drosophila* models of neuronal BOD1 deficiency by knocking down the fly ortholog of BOD1, CG5514, specifically in postmitotic neurons. All three models show specific deficits in habituation, an evolutionary conserved form of adaptive learning. Habituation is an important neuronal filtering mechanism, preventing information overload, and a prerequisite for higher cognitive functioning [43–45]. The *Drosophila* data thus provide independent evidence for a role of BOD1 in cognitive processes. It is also interesting to note that expression of the *PLK1* fly ortholog *polo* compares well with our finding of very low PLK1 expression in human brain tissues. While *Drosophila* polo is expressed in larval neuroblasts [46], polo transcripts seem to be absent in adult brain [47], supporting an evolutionarily conserved, PLK1-independent postmitotic function of BOD1 in humans and *Drosophila*.

The synapse is a key compartment of postmitotic, terminally differentiated neurons that plays a pivotal role in the maturation and maintenance of cognitive abilities. While the precise localisation of Bod1 at synapses warrants further investigation, synaptic localisation in mammalian neurons and the *Drosophila* synaptic phenotype upon strong knockdown suggest a novel role for Bod1 in synapse biology. This assumption is supported by the fact that *Drosophila* BOD1 (CG5514) is one of 893 Drosophila genes that were predicted to be involved in synapse assembly and function [48]. Interestingly, it has been shown that functional PP2A holoenzymes are required for synaptic growth and synaptic function at the Drosophila NMJ [49]. In view of our finding that BOD1 is required for proper PP2A function in human cells, this could mean that BOD1 is necessary for appropriate PP2A holoenzyme function and might thus be involved in the normal development and maintenance of cognitive features. PP2A has already been implicated in the aetiology of ID. Increased levels of the PP2A catalytic subunit were found in a cellular model of Fragile X syndrome [50] and PP2A was observed to dephosphorylate the Fragile X protein, FMRP, in immediate response to immediate group I metabotropic glutamate receptor (mGluR) stimulation [51]. What is more, putatively causative missense changes in PP2A subunits were found in several affected individuals from a large scale study of the genetics of developmental disorders [52] and most recently *de novo* missense mutations were identified in B56δ—and Aα—subunit of PP2A in individuals with ID [53].

BOD1 can also be linked to Intellectual Disability through a potential involvement in chromatin modification. The protein was recently found associated with SET1B complexes, COMPASS-like H3K3 histone methyltransferase multisubunit complexes, also containing HCFC1 [24], implicated in X-linked Intellectual Disability [25,26]. Like homozygous carriers of BOD1 mutations, individuals affected by HCFC1 mutations show no microcephaly phenotype. Furthermore MLL1, MLL2 and MLL3, the core subunits of other COMPASS complexes that share a number of subunits with SET1B, and might thus be affected indirectly by loss of BOD1, have been implicated in ID [54–57].

Although our data strongly indicate a post-mitotic role of Bod1, subtle mitotic defects in neurogenesis may also contribute to the cognitive impairment of individuals carrying *BOD1* mutations. For example, orientation of the cleavage plane is known to be important for cell fate choice during neurogenesis and development of the neural tube [58–60]. Accelerated progression into anaphase, analogous to those observed in *BOD1*^*-/-*^ Fibroblasts, may interfere with the correct positioning of the cleavage plane and lead to abnormalities in the generation of the correct proportions of neurons and progenitors.

Taken together our results identified homozygous loss of *BOD1* as a novel cause of ID and revealed a so far unappreciated postmitotic, likely synaptic function of BOD1. Thus our work opens up interesting avenues of research into the function of centrosomal proteins in fully differentiated neurons.

## Materials and Methods

### Ethics statement

The ID study was performed in agreement with the approval (2100) of the ethics committee of the Charité University Medicine Berlin ("Ethikkommission der Charité—Universitätsmedizin Berlin"), Germany. Written consent for the intellectually disabled individuals was provided by their parents.

### Sample collection, DNA and cell line preparation

The pedigree of the family reported here is shown in Fig 1A. Blood samples for DNA preparation were collected from the parents and all children of both branches and genomic DNA was extracted using a standard method. For the index patient (V:2), Fragile X was excluded by PCR and Southern blot analysis. Filter-dried blood of the index patient was screened by tandem mass spectrometry to exclude disorders of the amino acid, fatty acid (e.g. phenylketonuria) or organic acid metabolism [61,62]. Standard 450 G-band karyotyping was performed in order to exclude cytogenetically visible chromosomal aberrations.

An additional blood sample from patient V:2 was used to establish an EBV transformed lymphoblastoid cell line and skin biopsies were taken from individuals V:2 and V:3 to isolate and culture fibroblast cells.

### Whole genome linkage analysis and mutation screening

Genotyping was performed with Human Mapping 10K Array Version 2 (Affymetrix). Multipoint parametric linkage analysis was performed using Allegro [63] applying an autosomal recessive pattern of inheritance and disease allele frequency of 0.001. Sanger sequencing was performed and the whole coding and exon-flanking regions in the interval were screened (The primer sequences are available upon request). The resulting sequences were analyzed using the CodonCode aligner software.

### mRNA assays

Total RNA from the fibroblast and EBV transformed lymphoblast cultures was isolated by using TRIzol reagent RNA extraction protocol (Invitrogen). In addition, commercially available RNA (BioCat) was used from various tissues as indicated in Fig 3.

Semiquantitative RT-PCR was performed in two steps, after Dnase treatment of the RNA with RQ1 RNase-Free DNase (Promega), cDNA was synthesized with SuperScriptIII reverse transcriptase kit (Invitrogen) together with random hexamers and followed by Polymerase Chain Reaction for 35 cycles with primers in both sides of the gene (Forward primer:CATCGTGGAGCAGCTCAAG and Reverse primer:GCACTCTTATGTAACCGAATC) to amplify all the possible splice variants. Subsequently each of the splice variants were verified by isoform specific RT-PCR (The primer sequences are available upon request) as well as Sanger sequencing.

Isoform specific quantitative PCR was performed in 20 μl volumes and carried out in the ABI PRISM 7900 HT Sequence Detection System using a 96-well format. Cycling parameters: 10 min at 95oC followed by 40 cycles of 15s 95oC and 1 min 55oC. Amplification plot and predicted threshold cycle (Ct) values were obtained with the sequence Detection Software (SDS 2.1, PE Applied Biosystems).

We used absolute quantification to quantify unknown samples by interpolating their quantity from a standard curve. To construct the standard curve four-fold dilutions (1, 0.5, 0.25, 0.125) of a total RNA preparation from control cDNA were used. Negative controls (“no template control”, NTC) were used to verify amplification quality and to exclude contamination or primer-dimer artifacts. The gene used for normalising expression was *GAPDH*.

### NMD analysis

The fibroblast cells from two individuals affected by ID (V:2 and V:3) and two controls were cultured into two sister flasks, one treated with cycloheximide (CHX) (Sigma) at the concentration of 500 μg/ml and the other one non-treated (just added DMSO). After 7.5 hrs of incubation in 37C / 5%CO2, the cells were harvested, RNA was extracted and isoform specific quantitative PCRs were carried out.

### Cell lines and cell culture

HeLa S3 cells were maintained in EMEM (Lonza), Primary Fibroblasts were maintained in Quantum 333 media (PAA) or FibroPlus 333 (Capricorn-Scientific) and EBV-transformed lymphoblasts were maintained in RPMI 1640 media (Gibco). All media was supplemented with 10% FCS, 2 mM L-glutamine, 100 U/ml penicillin and 100 ug/ml streptomycin. Quantum 333 and FibroPlus 333 media was also supplemented with 10 ng/ml bFGF (Cell Signalling Technology). Cell lines were maintained at 37°C with 5% CO_2_ in a humidified incubator.

Fibroblast cell lines were electroporated using the Neon Transfection system (Life Technologies) as per the manufacturer’s instructions using a single pulse of 20 ms and 1600 V. Medium GC content control siRNA and BOD1 Stealth siRNA (5’-GCCACAAAUAGAACGAGCAAUUCAU-3’) were supplied by Life Technologies. The specificity of these reagents and absence of off-target effects were reported previously [16,17]. Briefly, BOD1 Stealth siRNA does not target Bod1L or Bod1L2 mRNA and all phenotypes associated with the BOD1 Stealth siRNA can be rescued using exogenous siRNA-resistant BOD1 expression constructs.

Unless otherwise indicated RO3306 was used at 9 μM, Monastrol at 100 nM and BI 2536 at 12.5 to 200 nM.

### Human fibroblast immunostainings and microscopy

Time-lapse imaging and fluorescence microscopy was performed as described [16]. Non-adherent lymphoblast cells were gently centrifuged onto the surface of a Labtek imaging chamber and constricted close to the coverslip surface by the addition of 50% Matrigel (BD Biosciences). During DIC timelapse imaging of lymphoblast and fibroblast cell lines 10 z sections, 3 μm apart were taken every 4 min for 18 hr on a DeltaVision Spectris (Applied Precision) fitted with an environment chamber (Solent) maintained at 37°C. All images were stored and manipulated using OMERO [64] or Photoshop (Adobe). Images were quantified using OMERO and m-tools as described [17]. Statistical significance was determined using Mann-Whitney Rank Sum Tests. Nuclear envelope breakdown (NEB) was defined as the time when a clear delineation of the nuclear envelope was no longer visible and the volume occupied by chromosomes began to expand. While not an exact measure of NEB, this approach combined with the 4 min interval between images provided sufficient accuracy to reveal differences in mitotic timing in these cell lines.

Rabbit anti-BOD1 [16] and mouse anti-B56α (BD Biosciences) were used at 1:100. Mouse anti-PLK1 (Upstate), and alpha-tubulin (Sigma) were used at 1:500. Human ACA (CREST) autoantisera (a kind gift from Sara Marshall, Ninewells Hospital, Dundee), were used at 1:1000. All fluorescently labelled secondary antibodies were obtained from Jackson ImmunoResearch Laboratories.

### RNA-sequencing

RNA Expression profiling was carried out on a SOLiD5500XL Sequencing platform as previously described [65], using RNA preparations form human embryonic stem cells (hES), induced pluripotent stem cells (iPSC) and commercially available RNA (BioCat) from various adult brain tissues as indicated in Fig 3.

### Isolation of murine primary corticoneuronal cells

The cortex was extracted from mouse embryonic brain at an age of E14.5. One day prior to sacrificing the mother plates were prepared as follows: Sterilized cover slips were placed tissue culture wells (12-well plates). The wells were then coated by incubating them for 30 min at room temperature or over night at 4°C with poly-D-Lysine/Laminin (poly-D-lysine 1:500 and laminin 1:50 in PBS, 1 ml per well). The wells were then washed twice with 1 ml PBS before adding 2 ml neurobasal medium to each well and then incubating at 37°C (8% CO_2_).

The pregnant mother was sacrificed by neck fracture, then the embryos were removed from the womb and placed in a sterile Petri dish containing PBS.

The preparation of corticoneuronal cells was carried out on a sterile flow bench. Embryos were briefly washed with PBS and then transferred in a fresh dry Petridish. Next the heads were removed and the brains excised. The brains were suspended in 1–2 ml DMEM, each. The Brain stem and brain lobes were separated under a stereoscope and the cortex removed, taking care to separate the cortex from the hippocampus. Cortex halves were then transferred in a chilled DMEM-Tube on ice. Next, single cells from isolated embryonic cortices were prepared: 5 ml trypsin was added and incubated for 7 min at 37°C (5%CO_2_), swirling once in between. Trypsin activity was then stopped by adding DMEM + 10% FCS and centrifugation (200xg for 3 min). This step was repeated twice. Finally the DMEM was carefully removed and 2 ml neurobasal medium were added, before decollating the cells using a narrowed pasteur pipette. Cells were then diluted by adding 28 ml neurobasal medium, counted and seeded to a density of 1.5x105 cells per cm^2^ in the prepared 12 well plates. After approximately 1 hr of incubation at 37°C (8% CO_2_) the medium was changed in order to remove unwanted cells. The next change of medium was after day 3–4. Subsequently the medium was changed every 7^th^ day. On the 7^th^ day after seeding (or later) cells were used for experimental applications.

### Transient transfection of murine primary cortical neurons

Cells were transfected by pEGFP-BOD1 (wt) or pEGFP-N1 vector (Control) at days 7 and 9 after preparation. A solution-A (containing 0.1 μg of plasmid DNA and 100 μl OptiMEM), and a solution-B (with 2 μl lipofectamine and 100 μl OptiMEM) were prepared and kept for 5 min at room temperature. Subsequently, solutions were mixed and incubated at room temperature for 20 min. Meanwhile, cell culture medium was replaced by transfection medium (antibiotic free culture medium). The transfection mixture was slowly added to the cells which then were incubated at 37°C (8% CO_2_). On both days cells were fixed with PFA 4% at two time points (6 and 8 hr).

### Fly stocks

Fly stocks were kept on standard *Drosophila* diet (cornmeal/sugar/yeast) at 25°C, in a12h:12h light/dark cycle. For the habituation experiments, flies were reared and tested at 25°C and 70% humidity, 28°C and 60% humidity was used during synapse morphology experiments and to generate ubiquitous RNAi-mediated knockdown for quantitative PCR (qPCR). Inducible RNAi lines against the *BOD1 Drosophila* ortholog CG5514 (vdrc105166, vdrc27445) and their corresponding genetic background control lines (vdrc60100, vdrc60000) were obtained from Vienna *Drosophila* RNAi Center [66]. A nonsense RNAi line, targeting a *C*.*elegans*-specific gene, was obtained from K. Keleman. Inducible short-hairpin RNAi line HMS00720 (BL32928) and its corresponding genetic background control line *y v; attP2*, *y+* (BL36303), generated by Transgenic RNAi Project (TRiP), were obtained from Bloomington Drosophila Stock Center. The ubiquitous actin-Gal4 driver *w*^*1118*^*; P(w[+mC] = Act5c-Gal4)/CyO*, also obtained from Bloomington *Drosophila* Stock Center, was used to generate RNAi-mediated knockdown for qPCR. In-house assembled panneuronal elav-Gal4 driver lines were used for habituation (2xGMR-wIR; elav-Gal4, UAS-Dicer2)) and synapse morphology experiments (UAS-Dicer2; elav-Gal4). In all experiments, progeny of a cross between these Gal4-driver lines and the genetic background of the respective RNAi line was used as a control.

### Light-off jump habituation

The light-off jump habituation assay was performed as previously described [32] with minor adaptations. Briefly, 3–7 day old individual male flies (Bod1^vdrc105166^, Bod1^HMS00720^) or female virgin flies (Bod1^vdrc24445^) were tested for jump responses in two independent 16-unit light-off jump habituation systems. 32 flies (16-flies/system) were simultaneously exposed to series of 100 short (15ms) light-off pulses with a 1s interval between the pulses. The noise amplitude of wing vibration following every jump response was recorded for 500ms after the start of pulse and a carefully determined threshold was applied to annotate jump responses. Data were collected and analyzed by custom-made Labview Software (National Instruments). High initial jumping response to light-off pulse decreased with growing number of pulses and flies were considered habituated when they failed to jump in 5 consecutive trials (non-jump criterion). Habituation was scored as the number of trials required to reach the non-jump criterion (Trials To Criterion, TTC). Main effects of genotype (mutant vs control), day and system on log transformed TTC values were tested using linear model regression analysis (lm) in R statistical software (R version 3.0.0 (2013-04-03)). A nonsense RNAi line targeting a C.elegans-specific gene (KK library construct) was also tested and did not affect habituation.

### Analysis of *CG5514* mRNA levels from whole *Drosophila* larvae by qPCR

Total RNA from 3rd instar larvae (3 biological replicates) was isolated using RNeasy Lipid Tissue Mini Kit (Qiagen). During the isolation procedure, samples were treated with RNase-free DNase Set (Qiagen). RNA isolated from Bod1^HMS00720^ and its respective genetic background control was used directly for cDNA synthesis. RNA isolated from Bod1^vdrc105166^ and its respective genetic background control was further processed by Oligotex mRNA Mini Kit and purified PolyA+ RNA was used for cDNA synthesis. First strand cDNA synthesis was performed using iScript cDNA Synthesis Kit (Biorad). Gene expression was analysed by real-time PCR (7900HT Fast Real-Time PCR system, Applied Biosystems). PCR reactions were performed in a volume of 25μl containing 150 nM primers and GoTag Green Mastermix (Promega). Primer sequences used for amplification of *CG5514*: 5’-ACAACAGTGGGGAGCCAG-3’ and 5’- CCTGTGCTAGTCGTCTCCG-3’. The amplicon spans the HMS00720 cleave site and should effectively detect the initial cleavage step of RNA interference. To determine the efficiency of the initial cleavage step of long vdrc105166 siRNA hairpin, the primers were designed to detect the region within the 5’ cleavage fragment, and qPCR was perfomed on PolyA+ mRNA. This ensures that in case of successful initial cleavage, regions 5’ to the cleavage site would not be amplified in qPCR reaction ***[67]***. Pol II was used as reference gene, primer sequences: 5’- TCAGAGTCCGCGTAACACC-3’, 5’- TGGTCACAAGTGGCTTCATC-3’.

### Analysis of synaptic morphology

Wandering L3 larvae were dissected and fixed in 3.7% paraformaldehyde for 30 minutes. Males were selected for Bod1^vdrc105166^ and female larvae for the X-linked Bod1^vdrc24445^. Type 1b neuromuscular junctions (NMJs) at muscle 4 were visualized using the primary antibody anti-dlg1 (1:25, Developmental Studies Hybridoma Bank) in combination with the Zenon Alexa Fluor 568 Mouse IgG1 labelling kit (Invitrogen). NMJ images were acquired using a Leica automated high-content microscope. Individual synapses of segments A2 –A5 were imaged and quantified using an in-house developed Fiji-compatible macro. Synaptic parameters were compared between the RNAi line and the corresponding genetic background control line using a student’s T-test.

## Supporting Information

S1 Fig Result of parametric linkage analysis. (DOCX)

S2 Fig (A) Mitotic progression of WT and BOD1-/- Lymphoblasts. (B) Mitotic distribution of WT and BOD1-/- fibroblasts. (C) Cell cycle distribution of RPE1 cells transfected with CTR or BOD1 siRNA. (D) Localisation of BOD1 in mitotic RPE1 cells.
(DOCX)

S3 Fig Clustal Omega multiple sequence alignment of Drosophila Bod1 (DM), human Bod1L1 (Hs1L1), human Bod1 (Hs1), and human Bod1L2 (Hs1L2). The shorter human Bod1 and Bod1L2 proteins share 30% amino acid sequence identity with the Drosophila Bod1 protein.
(DOCX)

S1 Table Quantification of relative expression level of CG8949 upon RNAi-mediated knock-down. The ubiquitous actin-Gal4 driver w1118; P(w[+mC] = Act5c-Gal4)/CyO (Bloomington Drosophila Stock Center24) was used to generate RNAi-mediated knock-down for quantitative PCR. Primer sequences used for amplification of CG5514: 5’- -3’ and 5’- -3’. Pol II was used as reference gene, using primer sequences 5’- -3’ and 5’- -3’. Ct = threshold cycle, dCt = CtCG5514 –CtPol II, ddCt = dCtRNAi–dCtcontrol, p-value calculated with student’s t-test.
(DOCX)
