# Supplementary material for: BOD1 Is Required for Cognitive Function in Humans and Drosophila
Source: PLoS Genet. 2016 May 11;12(5):e1006022. doi: 10.1371/journal.pgen.1006022 (PMC4864283; doi:10.1371/journal.pgen.1006022)
Supplement: S1 Fig — (DOCX) [file pgen.1006022.s001.docx]

_­­_

###

### S1 Fig: Result of parametric linkage Analysis
