# Supplementary material for: BOD1 Is Required for Cognitive Function in Humans and Drosophila
Source: PLoS Genet. 2016 May 11;12(5):e1006022. doi: 10.1371/journal.pgen.1006022 (PMC4864283; doi:10.1371/journal.pgen.1006022)
Supplement: S2 Fig — (A) Mitotic progression of WT and BOD1-/- Lymphoblasts. (B) Mitotic distribution of WT and BOD1-/- fibroblasts. (C) Cell cycle distribution of RPE1 cells transfected with CTR or BOD1 siRNA. (D) Localisation of BOD1 in mitotic RPE1 cells. (DOCX) [file pgen.1006022.s002.docx]

_­­­_

### S2 Fig: (A) Mitotic progression of WT and BOD1-/- Lymphoblasts. (B) Mitotic distribution of WT and BOD1-/- fibroblasts. (C) Cell cycle distribution of RPE1 cells transfected with CTR or BOD1 siRNA. (D) Localisation of BOD1 in mitotic RPE1 cells.
