# Supplementary material for: BOD1 Is Required for Cognitive Function in Humans and Drosophila
Source: PLoS Genet. 2016 May 11;12(5):e1006022. doi: 10.1371/journal.pgen.1006022 (PMC4864283; doi:10.1371/journal.pgen.1006022)
Supplement: S3 Fig — The shorter human Bod1 and Bod1L2 proteins share 30% amino acid sequence identity with the Drosophila Bod1 protein. (DOCX) [file pgen.1006022.s003.docx]

_­­_CLUSTAL O(1.2.1) multiple sequence alignment

Dm ------------------------------------------------------------ 0

Hs1L1 MATNPQPQPPPPAPPPPPPQPQPQPPPPPPGP-----------------GAGPGAG---- 39

Hs1 --------------------------MADGGGGGGTGAVGGGGTSQASAGAATGATGASG 34

Hs1L2 --------------------------MADGGGGGSG-------------GAGPASTRASG 21

Dm -------------MDDFIKTLIAEVKSQGVFDEFRFNCCLADVDTKPAYQNVRTQVETAV 47

Hs1L1 ---GAGGAGAGAGDPQLVAMIVNHLKSQGLFDQFRRD-CLADVDTKPAYQNLRQRVDNFV 95

Hs1 GGGPINPASLPPGDPQLIALIVEQLKSRGLFDSFRRD-CLADVDTKPAYQNLRQKVDNFV 93

Hs1L2 GGGPINPASLPPGDPQLIAIIVGQLKSRGLFDSFRRD-CKADVDTKPAYQNLSQKADNFV 80

::: :: .:**:*:**.** : * ***********: :.:. *

Dm NDFLAKQQWTPETNKVQLRERLRKHLMDSDVLDKGVDQIVDQVVNPKVATIFEPKIESIV 107

Hs1L1 ANHLATHTWSPHLNKNQLRNNIRQQVLKSGMLESGIDRIISQVVDPKINHTFRPQVEKAV 155

Hs1 STHLDKQEWNPTMNKNQLRNGLRQSVVQSGMLEAGVDRIISQVVDPKLNHIFRPQIERAI 153

Hs1L2 STHLDKQEWNPPANDNQLHDGLRQSVVQSGRSEAGVDRISSQVVDPKLNHIFRPQIEQII 140

.* .: *.* *. **:: :*: ::.* : *:*:* .***:**: *.*::* :

Dm YKYLGITPPARPTMLS-----------------APPLPPFGAHLNGSSLLNVETTVGLLP 150

Hs1L1 HEFLATLNHKEEGSGNTAPDDEKPDTSLITQGVPTPGPSANVANDAMSILETITS---LN 212

Hs1 HEFLAAQKKAAVPAPPPEPEGQ-----------DPPAPSQDTS----------------- 185

Hs1L2 HEFLVAQKEAAVPALPPEPEGQ-----------DPPAPSQDTS----------------- 172

:::* * * .

Dm TDLEQISPDSDRATVKS-ESRDDELPPG---------------------------VDDED 182

Hs1L1 QEASAARASTETSNAKTSERASKKLPSQPTTDTSTDKERTSEDMADKEKSTADSGGEGLE 272

Hs1 ------------------------------------------------------------ 185

Hs1L2 ------------------------------------------------------------ 172

Dm TSPSYELVSERK-----TLAIKEELNNVSLNNSDSVNGVSQASQLSQVSSDSRLTMASST 237

Hs1L1 TAPKSEEFSDLPCPVEEIKNYTKEHNNLILLNKDVQQES----------SEQKNKSTDKG 322

Hs1 ------------------------------------------------------------ 185

Hs1L2 ------------------------------------------------------------ 172

Dm ESMVDSHQHTAAHNSGEPENICEEAQMPKFSENSWDASAGTGR-------SEGRQLHFDI 290

Hs1L1 EKK------PDSNEKGERKKEKKEKTEKKFDHSKKSEDTQKVKDEKQAKEKEVESLKLPS 376

Hs1 ------------------------------------------------------------ 185

Hs1L2 ------------------------------------------------------------ 172

Dm ---KQDAITFEGTERKNSVSETTSTGLQILSIEDEIMSEVKANIDDANNASIE--SIAPE 345

Hs1L1 EKNSNKAKTVEGTKEDFSLIDSDVDGLTDITVSSVHTSDLSSFEEDTEEEVVTSDSMEEG 436

Hs1 ------------------------------------------------------------ 185

Hs1L2 ------------------------------------------------------------ 172

Dm PVQVPAPP--KVNPPPPPRPA-----SPKVEPPPPAPPGVES------------------ 380

Hs1L1 EITSDDEEKNKQNKTKTQTSDSSEGKTKSVRHAYVHKPYLYSKYYSDSDDELTVEQRRQS 496

Hs1 ------------------------------------------------------------ 185

Hs1L2 ------------------------------------------------------------ 172

Dm ---------------------------------PPGPQPPASPRFDPPPPHTIEPPPPPA 407

Hs1L1 IAKEKEERLLRRQINREKLEEKRKQKAEKTKSSKTKGQGRSSVDLEESSTKSLEPKAARI 556

Hs1 ------------------------------------------------------------ 185

Hs1L2 ------------------------------------------------------------ 172

Dm PPTLVPPPPPA-PPTIKPPPPPAPPTVEPPPPPPPAPPTVEPPPPPPPAPTKVEPPPPPA 466

Hs1L1 KEVLKERKVLEKKVALSKKRKKDSRNVEENSKK---KQQYEEDS-----KETLK---T-- 603

Hs1 ------------------------------------------------------------ 185

Hs1L2 ------------------------------------------------------------ 172

Dm PAEVEPPPPPAPTELE---PPPPPAPPKVELPPPP---APPKAEAAITPR-RAKGSNGFI 519

Hs1L1 SEHCEKEKISSSKELKHVHAKSEPSKPARRLSESLHVVDENKNESKLEREHKRRTSTPVI 663

Hs1 ------------------------------------------------------------ 185

Hs1L2 ------------------------------------------------------------ 172

Dm TELAVTPKESETRDKPHNVNTDVKEDEQPATT------------ED-------------- 553

Hs1L1 ME--GVQEETDTRDVKRQVERSEICTEEPQKQKSTLKNEKHLKKDDSETPHLKSLLKKEV 721

Hs1 ------------------------------------------------------------ 185

Hs1L2 ------------------------------------------------------------ 172

Dm --------KQDKP----LELGLDAPKDAVSTASESTESPTSTSSQSKSHSKSRDKEKEKD 601

Hs1L1 KSSKEKPEREKTPSEDKLSVKHKYKGDCMHKTGDETELHSSE----KGL-KVEENIQKQS 776

Hs1 ------------------------------------------------------------ 185

Hs1L2 ------------------------------------------------------------ 172

Dm RRHHRHSDDKHRRRSTD---------------------------------RDRDRSRDKS 628

Hs1L1 QQTKLSSDDKTERKSKHRNERKLSVLGKDGKPVSEYIIKTDENVRKENNKKERRLSAEKT 836

Hs1 ------------------------------------------------------------ 185

Hs1L2 ------------------------------------------------------------ 172

Dm HSKHSSSSSSKHSSSNSSSSKHKSSSSKNDKSSSSSSSRSNRESSSSKRSGTTSSSRHES 688

Hs1L1 KAEHKSR--------------------------RSSDSKIQKDSLGSKQHGITLQRRSES 870

Hs1 ------------------------------------------------------------ 185

Hs1L2 ------------------------------------------------------------ 172

Dm SSHKKHKSSSSSSRSERDKGKEKDKEREKDSQSRSHHSSSSSSSSSRRKDHDRGRDRDRN 748

Hs1L1 YSEDKCDMDSTNMDSNL------------------------------KPEEVVHKEKRRT 900

Hs1 ------------------------------------------------------------ 185

Hs1L2 ------------------------------------------------------------ 172

Dm KSNTSGSAENKAIHDDHSESKEKYKQRRGS-DSNDEGKPPSS--GGPAK--NSQPEDSAA 803

Hs1L1 ----KSLLEEKLVLKSKSKTQG--KQVKVVETELQEGATKQATTPKPDKEKNTEENDSEK 954

Hs1 ------------------------------------------------------------ 185

Hs1L2 ------------------------------------------------------------ 172

Dm ---TMKSDAPVENAN-----GTNGNSNGSTNGACDNVSGVVIVSDILQ-----QSTSSFV 850

Hs1L1 QRKSKVEDKPFEETGVEPVLETASSSAHSTQKDSSHRAKLPLAKEKYKSDKDSTSTRLER 1014

Hs1 ------------------------------------------------------------ 185

Hs1L2 ------------------------------------------------------------ 172

Dm ELTAGSQSHDR-------AESSKHEPEDIDGKEADNQPEKTEL-------EARQDECASQ 896

Hs1L1 KLSDGHKSRSLKHSSKDIKKKDENKSDDKDGKEVDSSHEKARGNSSLMEKKLSRRLCENR 1074

Hs1 ------------------------------------------------------------ 185

Hs1L2 ------------------------------------------------------------ 172

Dm NEVPTVE----PQTL------------------ADSVPDLPAKESMDTLEDEKVETNVEE 934

Hs1L1 RGSLSQEMAKGEEKLAANTLSTPSGSSLQRPKKSGDMTLIPEQEPMEIDSEPGVENVFEV 1134

Hs1 ------------------------------------------------------------ 185

Hs1L2 ------------------------------------------------------------ 172

Dm NKSE--------EESKP------------ENPPEECVDEPAQVGDVEDPPQDADKPATPV 974

Hs1L1 SKTQDNRNNNSQQDIDSENMKQKTSATVQKDELRTCTADSKATAPA-------YKPGRGT 1187

Hs1 ------------------------------------------------------------ 185

Hs1L2 ------------------------------------------------------------ 172

Dm PISNEQSDEFSADFVTHFEENTDEFRTRLQLINQ---LIEDRKNLLNRLSEDGTQEEAVD 1031

Hs1L1 GVNSN-S-EKHADHR-------STLTKKMHIQSAVSKMNPGEKEPI----HRGTTEVNID 1234

Hs1 ------------------------------------------------------------ 185

Hs1L2 ------------------------------------------------------------ 172

Dm IRALRRSLSKR----------RRSSMQEQHRVERETT----PPQPRSPSSSSTAGSP--- 1074

Hs1L1 SETVHRMLLSAPSENDRVQKNLKNTAAEEHVAQGDATLEHSTNLDSSPSLSSVTVVPLRE 1294

Hs1 ------------------------------------------------------------ 185

Hs1L2 ------------------------------------------------------------ 172

Dm --------------------------------AKRLRQD--------------EPKSSPT 1088

Hs1L1 SYDPDVIPLFDKRTVLEGSTASTSPADHSALPNQSLTVRESEVLKTSDSKEGGEGFTVDT 1354

Hs1 ------------------------------------------------------------ 185

Hs1L2 ------------------------------------------------------------ 172

Dm PSDASINSKENEALEKQEHAV--------------------------------------- 1109

Hs1L1 PAKASITSKRHIPEAHQATLLDGKQGKVIMPLGSKLTGVIVENENITKEGGLVDMAKKEN 1414

Hs1 ------------------------------------------------------------ 185

Hs1L2 ------------------------------------------------------------ 172

Dm **//**----------------- 1109

**S3 Fig:** Clustal Omega multiple sequence alignment of Drosophila Bod1 (DM), human Bod1L1 (Hs1L1), human Bod1 (Hs1), and human Bod1L2 (Hs1L2). The shorter human Bod1 and Bod1L2 proteins share ͂30% amino acid sequence identity with the Drosophila Bod1 protein.

Hs1L1 **//**TRGQQRVEEAPVKKAKR 3051

Hs1 **//**----------------- 185

Hs1L2 **//**----------------- 172
