# Supplementary material for: BOD1 Is Required for Cognitive Function in Humans and Drosophila
Source: PLoS Genet. 2016 May 11;12(5):e1006022. doi: 10.1371/journal.pgen.1006022 (PMC4864283; doi:10.1371/journal.pgen.1006022)
Supplement: S1 Table — The ubiquitous actin-Gal4 driver w1118; P(w[+mC] = Act5c-Gal4)/CyO (Bloomington Drosophila Stock Center24) was used to generate RNAi-mediated knock-down for quantitative PCR. Primer sequences used for amplification of CG5514: 5’- -3’ and 5’- -3’. Pol II was used as reference gene, using primer sequences 5’- -3’ and 5’- -3’. Ct = threshold cycle, dCt = CtCG5514 –CtPol II, ddCt = dCtRNAi–dCtcontrol, p-value calculated with student’s t-test. (DOCX) [file pgen.1006022.s004.docx]

| **Genotype** | **dCTav** | **Sd** | **ddCT** | **% rel. expr.** | **p-val.** |
| --- | --- | --- | --- | --- | --- |
| ***CG5514^vdrc105166^*** | 2.49 | 0.25 | 2.13 | 23 | 0.001 |
| **control** | 0.36 | 0.38 |  |  |  |
| ***CG5514^vdrcHMS00720^*** | 3.41 | 0.31 | 1.42 | 31 | 0.009 |
| **control** | 1.99 | 0.05 |  |  |  |

**S1 Table:** Quantification of relative expression level of CG8949 upon RNAi-mediated knock-down. The ubiquitous actin-Gal4 driver w1118; P(w[+mC]=Act5c-Gal4)/CyO (Bloomington Drosophila Stock Center24) was used to generate RNAi-mediated knock-down for quantitative PCR. Primer sequences used for amplification of CG5514: 5’- -3’ and 5’- -3’. Pol II was used as reference gene, using primer sequences 5’- -3’ and 5’- -3’. Ct = threshold cycle, dCt = CtCG5514 – CtPol II, ddCt = dCtRNAi – dCtcontrol, p-value calculated with student’s t-test.
